# Supplementary material for: End Sequence Analysis Toolkit (ESAT) expands the extractable information from single-cell RNA-seq data
Source: Genome Res. 2016 Oct;26(10):1397–410. doi: 10.1101/gr.207902.116 (PMC5052061; doi:10.1101/gr.207902.116)
Supplement: Supplemental Material [file supp_gr.207902.116_Supp_Materials.tar.gz › ESAT-esat_v0.1/doc/html/classbroad_1_1core_1_1siphy_1_1_conserved_p_w_m_scanner.html]

ESAT: broad.core.siphy.ConservedPWMScanner Class Reference


ESAT


- Main Page
- Related Pages
- Packages
- Classes
- Files

- Class List
- Class Index
- Class Hierarchy
- Class Members

All Classes Namespaces Files Functions Variables Enumerator Pages

- broad
- core
- siphy
- ConservedPWMScanner

Public Member Functions |
Protected Member Functions |
List of all members

broad.core.siphy.ConservedPWMScanner Class Reference

Collaboration diagram for broad.core.siphy.ConservedPWMScanner:

[legend]

|  |  |
| --- | --- |
| Public Member Functions | |
|  | ConservedPWMScanner (String pwmFile) throws IOException, ParseException |
|  | |
|  | ConservedPWMScanner (List< PositionWeightMatrix > matrices) |
|  | |
| Iterator< PositionWeightMatrix > | getPWMiterator () |
|  | |
| void | setBackgroundModel (AnnotationReader<?extends GenomicAnnotation > reader, Map< String, Sequence > org) throws IOException |
|  | |
| void | setModel (EvolutionaryModel model) |
|  | |
| int | getAlignmentChunkSize () |
|  | |
| void | setAlignmentChunkSize (int alignmentChunkSize) |
|  | |
| Map< PositionWeightMatrix,   GenomicAnnotation > | scan (MAFIO chrMafIO, List< BED > annotations, int shuffles, float seedMinScore) throws IOException, ParseException |
|  | |
| void | setIgnoreList (List< String > ignoreList) |
|  | |

|  |  |
| --- | --- |
| Protected Member Functions | |
| List< BED > | slidePWM (PositionWeightMatrix pwm, double minSeedScore, short[] encodedRef, List< int[]> ungappedChunks, int numPermutations) |
|  | |

## Constructor & Destructor Documentation

|  |  |  |  |  |  |
| --- | --- | --- | --- | --- | --- |
| broad.core.siphy.ConservedPWMScanner.ConservedPWMScanner | ( | String | *pwmFile* | ) | throws IOException, ParseException |

|  |  |  |  |  |  |
| --- | --- | --- | --- | --- | --- |
| broad.core.siphy.ConservedPWMScanner.ConservedPWMScanner | ( | List< PositionWeightMatrix > | *matrices* | ) |  |

## Member Function Documentation

|  |  |  |  |  |
| --- | --- | --- | --- | --- |
| int broad.core.siphy.ConservedPWMScanner.getAlignmentChunkSize | ( |  | ) |  |

Here is the caller graph for this function:

|  |  |  |  |  |
| --- | --- | --- | --- | --- |
| Iterator<PositionWeightMatrix> broad.core.siphy.ConservedPWMScanner.getPWMiterator | ( |  | ) |  |

|  |  |  |  |
| --- | --- | --- | --- |
| Map<PositionWeightMatrix, GenomicAnnotation> broad.core.siphy.ConservedPWMScanner.scan | ( | MAFIO | *chrMafIO*, |
|  |  | List< BED > | *annotations*, |
|  |  | int | *shuffles*, |
|  |  | float | *seedMinScore* |
|  | ) |  | throws IOException, ParseException |

Here is the call graph for this function:

|  |  |  |  |  |  |
| --- | --- | --- | --- | --- | --- |
| void broad.core.siphy.ConservedPWMScanner.setAlignmentChunkSize | ( | int | *alignmentChunkSize* | ) |  |

|  |  |  |  |
| --- | --- | --- | --- |
| void broad.core.siphy.ConservedPWMScanner.setBackgroundModel | ( | AnnotationReader<?extends GenomicAnnotation > | *reader*, |
|  |  | Map< String, Sequence > | *org* |
|  | ) |  | throws IOException |

|  |  |  |  |  |  |
| --- | --- | --- | --- | --- | --- |
| void broad.core.siphy.ConservedPWMScanner.setIgnoreList | ( | List< String > | *ignoreList* | ) |  |

|  |  |  |  |  |  |
| --- | --- | --- | --- | --- | --- |
| void broad.core.siphy.ConservedPWMScanner.setModel | ( | EvolutionaryModel | *model* | ) |  |

|  |  |  |  |
| --- | --- | --- | --- |
| List<BED> broad.core.siphy.ConservedPWMScanner.slidePWM | ( | PositionWeightMatrix | *pwm*, |
|  |  | double | *minSeedScore*, |
|  |  | short[] | *encodedRef*, |
|  |  | List< int[]> | *ungappedChunks*, |
|  |  | int | *numPermutations* |
|  | ) |  |  |

protected

Here is the call graph for this function:

Here is the caller graph for this function:

---

The documentation for this class was generated from the following file:

- java/broad/core/siphy/ConservedPWMScanner.java


---

Generated on Fri Aug 15 2014 15:10:56 for ESAT by  

 1.8.7
